# Assessment of apoptosis in human breast tissue using an antibody against the active form of caspase 3: relation to tumour histopathological characteristics

**DOI:** 10.1054/bjoc.2001.2115

**Published:** 2001-11

**Authors:** I Hadjiloucas, A P Gilmore, N J Bundred, C H Streuli

**Affiliations:** Departments of Surgery, School of Biological Sciences, University of Manchester, Manchester, UK

**Keywords:** apoptosis, caspase 3, breast cancer, mammary gland

## Abstract

Apoptosis is of important significance in the pathogenesis of cancer. Many methods are available for the measurement of apoptosis but the ‘gold standard’ is to identify apoptotic cells by their morphological features using microscopy. Caspase 3 is a cytosolic enzyme that is activated only in cells committed to undergo apoptosis. The activation of caspase 3 precedes the development of the classical morphological features of apoptosis. Using immunohistochemistry with an antibody against the active form of caspase 3, the apoptotic index (AI) was measured in 116 samples of human breast tissue (22 normal/benign and 94 invasive carcinomas). The AI obtained by measuring caspase activation has a strong correlation with the AI derived by morphological assessment (r = 0.736, *P* < 0.01). The AI is higher in the invasive group than in the benign group (*P* = 0.008), and in invasive cancer high AI is associated with high tumour grade (*P* = 0.013), positive node status (*P* < 0.001) and negative steroid receptor status (*P* = 0.001 for ER; *P* = 0.004 for PR). No significant association is observed between AI and tumour size. Measurement of apoptosis by immunohistochemistry using an antibody against the active form of caspase 3 is therefore reliable and correlates strongly with morphological assessment. © 2001 Cancer Research Campaign  http://www.bjcancer.com


*British Journal of Cancer* (2001) **85**(10), 1522–1526
© 2001 Cancer Research Campaign
doi: 10.1054/ bjoc.2001.2115, available online at http://www.idealibrary.com on IDEAL

http://www.bjcancer.com

### Assessment of apoptosis in human breast tissue using an antibody against the active form of caspase 3: relation to tumour histopathological characteristics

**I Hadjiloucas[1], AP Gilmore[2], NJ Bundred[1] and CH Streuli[2]**

Departments of [1]Surgery & [2]School of Biological Sciences, University of Manchester, Manchester, UK

**Summary** Apoptosis is of important significance in the pathogenesis of cancer. Many methods are available for the measurement of apoptosis but the 'gold standard' is to identify apoptotic cells by their morphological features using microscopy. Caspase 3 is a cytosolic enzyme that is activated only in cells committed to undergo apoptosis. The activation of caspase 3 precedes the development of the classical morphological features of apoptosis. Using immunohistochemistry with an antibody against the active form of caspase 3, the apoptotic index (AI) was measured in 116 samples of human breast tissue (22 normal/benign and 94 invasive carcinomas). The AI obtained by measuring caspase activation has a strong correlation with the AI derived by morphological assessment ($r = 0.736$, $P < 0.01$). The AI is higher in the invasive group than in the benign group ($P = 0.008$), and in invasive cancer high AI is associated with high tumour grade ($P = 0.013$), positive node status ($P < 0.001$) and negative steroid receptor status ($P = 0.001$ for ER; $P = 0.004$ for PR). No significant association is observed between AI and tumour size. Measurement of apoptosis by immunohistochemistry using an antibody against the active form of caspase 3 is therefore reliable and correlates strongly with morphological assessment. © 2001 Cancer Research Campaign http://www.bjcancer.com

**Keywords**: apoptosis; caspase 3; breast cancer; mammary gland

Apoptosis (programmed cell death) is essential during development and in the maintenance of tissue homeostasis, and serves to delete damaged or redundant cells (Thompson, 1995; Vaux and Korsmeyer, 1999). Disruption of the normal mechanism for apoptosis can result in a number of pathological conditions. The inappropriate survival and propagation of cells with damaged DNA may contribute to the development of neoplasia and metastases (Sarasin and Stary, 1997).

Apoptosis is regulated by a wide variety of survival signals including growth factors, cell adhesion, and by cellular mechanisms that monitor DNA integrity (Gilmore et al, 2000; Hengartner, 2000; Rich et al, 2000; Streuli and Gilmore, 1999). The absence of survival signals or sensing of DNA damage results in the activation of caspases, a family of cysteine proteases that cleave a wide variety of cellular proteins, ultimately leading to the destruction of the cell. Caspases are present in the cytoplasm as zymogens, and the initiation of apoptosis results in the cleavage of the pro-enzyme and the formation of a tetrameric molecule, the active form of the enzyme (Green, 1998; Ashkenazi and Dixit, 1999). Caspase 3 is a member of this family, which is present in a wide variety of cells and tissues including breast.

The apoptosis execution mechanism results in the development of characteristic morphological features, which include condensation of the nucleus and chromatin followed by cell shrinkage and the appearance of cytoplasmic blebs. The cell detaches from its neighbours and the nucleus fragments (Hall, 1999). DNA is cleaved into approximately 200 base pair fragments that aggregate to form well defined dense hyperchromatic bodies called 'apoptotic bodies', and are visible by conventional microscopy. The apoptotic cells are recognised by the cells of the reticuloendothelial system and are removed by phagocytosis (Savill and Fadok, 2000). The time taken from the activation of the apoptotic mechanism to cell clearance is approximately 1–3 hours, indicative of the dynamic nature of the process (Sanderson, 1976; Matter, 1979; Wyllie et al, 1980; Gavrieli et al, 1992; Hall, 1999).

The measure for quantification of apoptosis is the apoptotic index (AI). In an epithelial tissue or carcinoma line, the AI is a percentage expression of the ratio of the number of apoptotic events to the total number of the epithelial cells examined (Potten, 1996; Lipponen, 1999). Various methods are used for the detection of cells undergoing apoptosis and these methods include morphological assessment, in situ end labelling (ISEL), terminal-deoxynucleotidyl-transferase-mediated dUTP nick end labelling (TUNEL) and the use of a monoclonal antibody to single-stranded DNA (Frankfurt et al, 1997; Mainwaring et al, 1998). However, each of these methods has significant limitations. The quantification of apoptotic events by morphological assessment depends on the examiner's subjective assessment in order to accurately identify apoptotic cells. Accurate distinguishing features of apoptosis for morphological assessment also tend to be late events and are only apparent for a brief window of time, therefore leading to lower scores of apoptosis than actually occurs. TUNEL, on the other hand, fails to distinguish apoptosis from necrosis, and only detects late stages of the process, and furthermore can produce artefactual positive results where nuclei are cut during the sectioning procedure (Grasl-Kraupp et al, 1995; Frankfurt et al, 1997).

In this study, we have examined apoptosis in mammary epithelia by immunohistochemistry using an antibody specific for the active form of caspase 3. We compared this with the morphological

*Received 19 February 2001*
*Revised 18 July 2001*
*Accepted 23 July 2001*

method for the examination of apoptosis (Hall, 1999). Furthermore, we correlated apoptosis derived by both immunohistochemistry and morphological assessment with tumour histopathological characteristics. Our results demonstrate higher levels of apoptosis in invasive, node-positive, breast cancer than benign breast disease.

### MATERIALS AND METHODS

### Tissue

Samples of benign breast tissue and invasive breast carcinomas were randomly selected from the archives at the Manchester Breast Unit. Permission to collect normal breast tissue from patients undergoing surgery in the Unit was granted by the South Manchester Ethical Committee and all patients gave written informed consent. The histopathological characteristics for most of the tumours including histological type, size, grade and axillary lymph node status were available following the routine histological examination of the excised specimen immediately following surgery. Clinical information of these patients was also available from a large database. The tissue was embedded in paraffin and, using a microtome, serial consecutive sections 7 µm in thickness were obtained and mounted in APES-coated slides. One section from each sample was stained with haematoxylin and eosin for morphological assessment of apoptosis. Immunohistochemistry was performed on the next consecutive section.

### Immunohistochemical staining for active caspase 3

The sections were deparaffinised in xylene and rehydrated in ethanol of increasing dilution. The sections were then treated with 0.3% Triton-X100 for 15 minutes and non-specific binding sites were blocked with goat serum. The sections were washed with 0.1% bovine serum albumin (BSA) diluted in phosphate buffer saline (PBS). A rabbit polyclonal primary antibody against the active form of caspase 3 (R&D systems, Abingdon, Oxfordshire) was then applied to the sections at a concentration of 1:1000 diluted in goat serum for 1 hour at 37˚C in a moist chamber. Following three 5-minute washes with 0.1% BSA/PBS the sections were treated with 0.03% hydrogen peroxide containing sodium azide (DAKO EnVision+ System). The sections were washed once for 5 minutes and a peroxidase-labelled polymer conjugated to goat anti-rabbit secondary antibody (DAKO) was applied for 30 minutes at 37˚C. One 5-minute wash with 0.1% BSA/PBS and two 5-minute washes with plain PBS followed and the AEC (3-amino-9-ethylcarbazole)/substrate-chromogen (DAKO) was applied for 15 minutes. The sections were washed in distilled water and glass coverslips were mounted using glycerol. The negative controls were treated in the same way omitting the primary antibody, which was substituted with goat serum. For positive controls, sections from tumours known to have very high apoptotic activity were used.

### Assessment of apoptosis

The AI was calculated as a percentage of the apoptotic cells out of the total number of cells examined. The AI was obtained by morphological assessment of the H&E stained sections or by examination of the sections stained using immunohistochemistry. In the former assessment, the classical characteristics of apoptosis were used as the criteria for the identification of the apoptotic cells

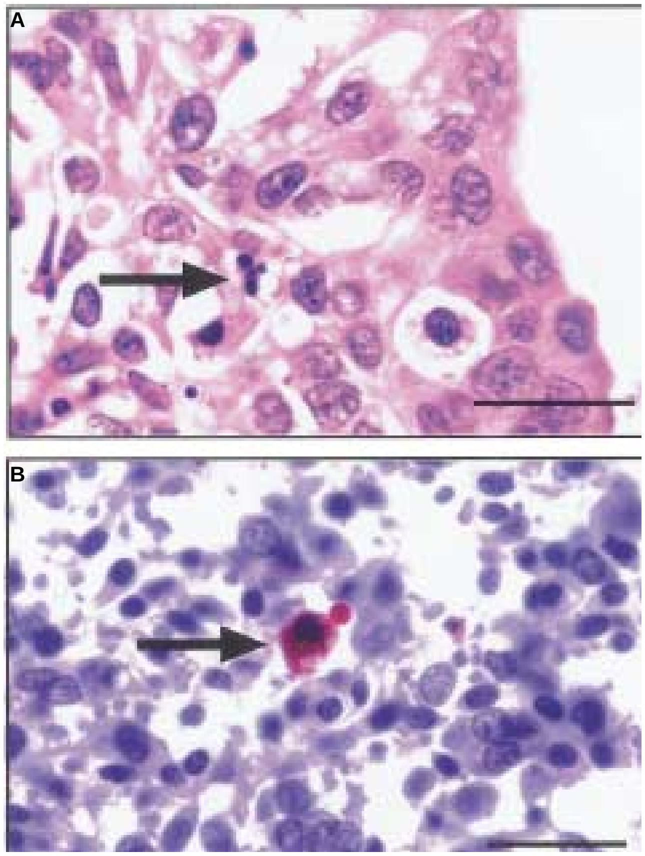

**Figure 1** (**A**) A section of invasive breast cancer stained with haematoxylin and eosin. The apoptotic cell (arrow) is identified by the presence of the rounded, densely stained 'apoptotic bodies' which represent parts of the fragmented nucleus. (**B**) A section of invasive breast cancer stained by immunohistochemistry with a primary antibody against the active form of caspase 3. The red orange staining of the apoptotic cell (arrow) makes its identification easier and in the latter all the identifiable cells exhibiting staining were counted (Figure 1). Control studies using primary cultures of mammary epithelial cells indicate almost complete coincidence of apoptosis measured by altered nuclear morphology and by immunostaining for active caspase 3 (data not shown). Apoptotic cells that were present in areas where intense necrosis had occurred were excluded from the analysis.

### Immunostaining for oestrogen receptor

Following deparaffinisation with xylene and blocking of the endogenous peroxidase activity by incubation with 0.3% hydrogen peroxide, non-specific binding was blocked with 20% normal rabbit serum (DAKO X902) for 15 minutes. Primary antibody (1:100 dilution of mouse monoclonal anti-human ER; DAKO M7047) was applied overnight at 4˚C. A biotinylated rabbit anti-mouse immunoglobulin (DAKO E413) was applied as the secondary antibody (1:350 dilution) for 1 hour. Streptavidin (DAKO) was applied for 30 minutes and sections were visualised with DAB (Abbott Diagnostics). Sections where 5% or more of cells showed staining for oestrogen receptor (ER) were considered as positive.

### Immunostaining for progesterone receptor

Following blocking of the non-specific binding and endogenous peroxidase activity, the section was treated with a rat monoclonal

*© 2001 Cancer Research Campaign*

antibody PgR-ICA (Abbott Diagnostics) at a dilution 1:4 overnight. Following rinsing, sections were incubated with biotinylated rabbit anti-rat antibody (1:100 dilution) for 30 minutes at room temperature. Sections were treated with Streptavidin for 30 minutes and visualised with DAB. Sections where 5% or more of cells showed staining for progesterone receptor (PR) were considered as positive.

### Light microscopy

All sections were examined within 48 hours of staining. A 40 × objective lens was used with a 10× eyepiece lens and at least 1000 epithelial cells were examined in every section. The same topographical areas were examined in consecutive sections of the same sample in order to minimise tissue heterogeneity. Areas of intense necrosis or apoptosis were excluded as this would have produced a non-representative result.

### Statistical analysis

All the statistical analysis of the results was performed using the statistical package for social science (SPSS) under the guidance of a professional statistician.

### RESULTS

22 samples of normal breast tissue (2 samples from reduction mammoplasty specimens and 20 samples from women undergoing surgery for benign breast disease) and 94 primary invasive breast carcinomas were examined in this study. The mean age group of the subjects was 57.3 years with a range from 16 to 84 years (Table 1).

To evaluate if there was a correlation between morphological and immunohistochemical analysis of apoptosis, AI values using both methods were obtained for every sample and plotted as a scatter diagram (Figure 2). The overall Spearman's correlation coefficient ($n$ = 116) between these 2 variables is 0.736 ($P$ value < 0.01). This indicates a strong correlation between the 2 methods and demonstrates that caspase staining produces similar apoptosis data to the method of morphological assessment.

To determine if apoptosis relates to breast cancer aggressiveness we measured the AI in tissue from 94 cancer patients and compared apoptosis levels amongst the known breast cancer prognostic indicators. First, the AI was lower in normal/benign breast tissue compared to the invasive group of samples, the difference in the AI values being statistically significant ($P$ = 0.008) (Figure 3A). Second, the median AI in the node-positive group of breast cancers was significantly ($P$ < 0.001) higher than the corresponding value in the node-negative group (Figure 3B). Third, grade III tumours had a higher AI than grade I or II tumours ($P$ = 0.013) (Figure 3C). Finally, a significantly higher AI ($P$ = 0.009) was found in the ER-negative group of tumours than the ER-positive group (Figure 3D). The same trend was observed with the progesterone receptor (AI = 0.54 for PgR negative vs 0.185 for PgR positive; $P$ = 0.05) (data not shown). Together these data indicate that substantially more apoptosis is detected in breast cancer than in the normal tissue.

### DISCUSSION

There are 2 important conclusions to be drawn from this study. The first is that, by taking advantage of the activation of caspase 3 during apoptosis, we have developed a robust method for assessing apoptosis in biopsies from breast epithelia. The second is

**Table 1** (a) Characteristic features of the normal/benign breast disease group. (b) Characteristic features of the tumour group

| | Number of specimens | |
|---|---|---|
| **a** | | |
| Type | | |
| Normal | 2 | |
| Fibroadenoma | 14 | |
| Fibrocystic disease | 4 | |
| Duct ectasia | 1 | |
| Sclerosing adenosis | 1 | |
| Apoptotic index | | |
| | Morph | IHC |
| Median | 0.165 | 0.085 |
| Interquartile range | 0.07–0.35 | 0–0.20 |
| **b** | | |
| Tumour type | | |
| Ductal | 80 | |
| Lobular | 6 | |
| Mixed | 2 | |
| Other | 6 | |
| Grade | | |
| I | 11 | |
| II | 23 | |
| III | 53 | |
| Node status | | |
| Positive | 43 | |
| Negative | 49 | |
| Size | | |
| < 20 mm | 41 | |
| 21–50 mm | 48 | |
| > 50 mm | 4 | |
| ER status | | |
| Positive | 54 | |
| Negative | 25 | |
| PR status | | |
| Positive | 46 | |
| Negative | 33 | |
| Apoptotic index | | |
| | Morph | IHC |
| Median | 0.285 | 0.33 |
| Interquartile range | 0.08–0.585 | 0–0.6575 |

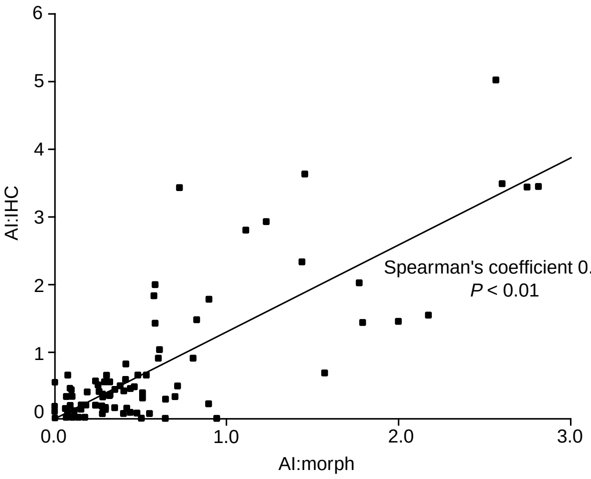

**Figure 2** Comparison of the morphological and immunohistochemical methods of apoptosis assessment. Parallel sections of all the tissue samples were scored for apoptosis both by immunostaining for activated caspase-3 (A1:IHC) 0.20 (0–0.55) and by using the conventional method of assessing nuclear morphology (AI:morph) 0.27 (0.08–0.54)

 *© 2001 Cancer Research Campaign*

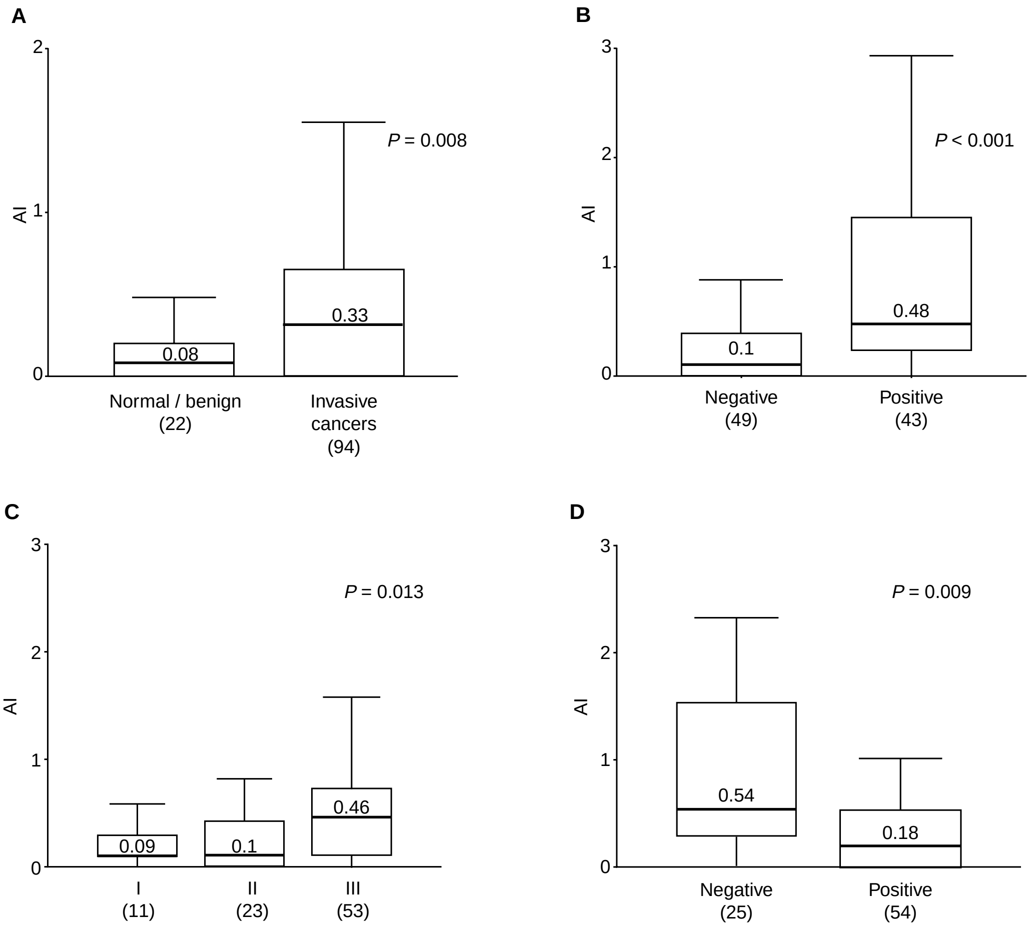

**Figure 3** (**A**) Comparison of the AI between normal/benign breast and invasive carcinoma. In each case, the number of samples analysed is shown in brackets below the bars, and the median AI value is shown within the bars. Similar results were obtained using both caspase-3 and morphological methods of apoptosis assessment. (**B**) Comparison of the apoptotic indices in the lymph node positive and negative groups of tumour tissue. (**C**) Comparison of the apoptotic indices between tumour grades. (**D**) Comparison between the oestrogen receptor (ER)-positive and-negative groups. Sections where more than 5% of cells exhibited ER staining were categorised as positive and those where less than 5% of cells exhibited staining as negative that significantly more apoptosis occurs in breast carcinoma than in normal or benign breast tissue and that apoptosis is higher in the more invasive cancers.

The appearance of the active form of caspase 3 in the cytoplasm of the cells undergoing apoptosis is an early event and precedes the development of the classical morphological features of apoptosis. Hence, immunohistochemistry using an antibody against the active form of caspase 3 detects cells undergoing apoptosis at an early stage. The bright orange staining of apoptotic cells in light microscopy, using this method, makes the detection of apoptotic cells in the section easy and less dependent on the observer's subjective opinion. Non-specific background staining was minimal in the protocol described. A very small number of cells, less than 0.01%, showed morphological features of apoptosis but no staining, and in this study they were not included in the analysis. A similar finding has also been observed in a previous study with TUNEL and in-situ nick translation, and is expected as some cells may undergo apoptosis without the activation of caspase 3 (Mainwaring et al, 1998).

The apoptotic indices obtained by the 2 methods show strong correlation, indicating that assessing apoptosis by immunohistochemistry using an antibody to the active form of caspase 3 is an accurate and reliable method. This method is sensitive, specific,

**Table 2** Comparison of our study with others measuring the apoptotic index AI in primary invasive breast carcinomas using different methods of assessment and different units of measurement. (a) Studies where AI is calculated as a percentage of the total number of cells counted. (b) Studies where AI is calculated per unit area of tissue examined

| | Author | Method | Unit | Mean AI |
|---|---|---|---|---|
| a | Frankfurt (1997) | Mab ss-DNA | (% Apoptotic cells/Total number of cells examined) | 5.5 |
| | Frankfurt (1997) | TUNEL | (% Apoptotic cells/Total number of cells examined) | 0.74 |
| | Vakkala (1999) | ISEL | (% Apoptotic cells/Total number of cells examined) | 0.74 |
| | Harn (1997) | ISEL | (% Apoptotic cells/Total number of cells examined) | 0.36 |
| | This study | active caspase-3 staining | (% Apoptotic cells/Total number of cells examined) | 0.33 |
| b | Rochaix (1999) | TUNEL | Apoptotic cells $mm^{-2}$ | 9.7 |
| | Lipponen (1994) | H&E | Apoptotic cells $mm^{-2}$ | 11.3 |
| | Zhang (1998) | H&E | Apoptotic cells $mm^{-2}$ | 11.0 |
| | Zheng (1998) | H&E | Apoptotic cells $mm^{-2}$ | 11.14 |

*© 2001 Cancer Research Campaign*

easy to apply and free of subjective interpretation, and therefore does not require an experienced observer trained to recognise apoptotic cells in tissue sections. It also does not rely on DNA fragmentation, a late event in the apoptotic process. Thus we recommend that this method should be adopted more widely in the study of apoptosis in breast cancer.

We have found that a high AI is associated with node positivity and oestrogen receptor negativity in breast cancer. The AI is highest in the poorly differentiated grade III tumours. Other studies have shown that higher apoptosis is associated with grade III tumours, strengthening the findings in this study (Lipponen et al, 1994; Frankfurt et al, 1997; Zheng and Zhan, 1998; Rochaix et al, 1999). We found no association between the AI and tumour size, supporting the results of 2 earlier studies (Lipponen et al, 1994; Berardo et al, 1998).

Our results compare well with other studies (Table 2), where high levels of apoptosis have also been associated with high tumour grade, lack of tubule formation, increased risk of lymph node metastasis, and loss of steroid receptors (Lipponen et al, 1994; Frankfurt et al, 1997; Zhang et al, 1998; Zheng and Zhan, 1998; Rochaix et al, 1999). In one study, the AI was shown to be associated with shorter recurrence-free survival (Lipponen et al, 1994). In another study, the AI was found to be a predictor of survival in univariate analysis but failed to do so in multivariate analysis. Recurrent tumours had significantly higher proliferation and apoptosis compared to primary tumours and correlated with reduced patient survival (Vakkala et al, 1999).

Together, these results demonstrate that AI is higher in carcinoma tissue than in benign breast epithelium. It is well known that proliferation rates increase in carcinoma, and the finding that apoptosis rates are also higher is suggestive of a tissue that is much more rapidly turning over. The increases in apoptosis may be indicative of substantial genomic instability that is manifested during breast cancer progression.

### ACKNOWLEDGEMENTS

CHS is a Wellcome Trust Senior Fellow in Basic Biomedical Science. APG is a Wellcome Trust Research Career Development Fellow. We are grateful to Dr Fiona Knox, Dr Elisabeth Anderson, Janine Oliver, Kai Chan, Anthony Valentijn and the Department of Medical Statistics at the University Hospital of South Manchester for their help with statistical analysis.

### REFERENCES

Ashkenazi A and Dixit VM (1999) Apoptosis control by death and decoy receptors. *Current Opin Cell Biol* **11**: 255–260

Berardo MD, Elledge RM, de Moor C, Clark GM, Osborne CK and Allred DC (1998) bcl-2 and apoptosis in lymph node positive breast carcinoma. *Cancer* **82**: 1296–1302

Frankfurt OS, Robb JA, Sugarbaker EV and Villa L (1997) Apoptosis in breast carcinomas detected with monoclonal antibody to single-stranded DNA: relation to bcl-2 expression, hormone receptors, and lymph node metastases. *Clin Cancer Res* **3**: 465–471

Gavrieli Y, Sherman Y and Bensasson SA (1992) Identification of programmed cell-death insitu via specific labeling of nuclear-dna fragmentation. *Journal of Cell Biology* **119**: 493–501

Gilmore AP, Metcalfe AM, Romer LH and Streuli CH (2000) Integrin-mediated survival signals regulate the apoptotic function of Bax through its conformation and subcellular localization. *J Cell Biol* **149**: 431–445

Grasl-Kraupp B, Ruttkay-Nedecky B, Koudelka H, Bukowska K, Bursch W and Schulte-Hermann R (1995) In situ detection of fragmented DNA (TUNEL assay) fails to discriminate among apoptosis, necrosis, and autolytic cell death: a cautionary note. *Hepatology* **21**: 1465–1468

Green DR (1998) Apoptotic pathways: the roads to ruin. *Cell* **94**: 695–698

Hall PA (1999) Assessing apoptosis: a critical survey. *Endocr Relat Cancer* **6**: 3–8

Harn HJ, Shen KL, Yueh KC, Ho LI, Yu JC, Chiu SC and Lee WH (1997) Apoptosis occurs more frequently in intraductal carcinoma than in infiltrating duct carcinoma of human breast cancer and correlates with altered p53 expression: detected by terminal-deoxynucleotidyl-transferase-mediated dUTP-FITC nick end labelling (TUNEL). *Histopathology* **31**: 534–539

Hengartner MO (2000) The biochemistry of apoptosis. *Nature* **407**: 770–776

Lipponen P (1999) Apoptosis in breast cancer: relationship with other pathological parameters. *Endocr Relat Cancer* **6**: 13–16

Lipponen P, Aaltomaa S, Kosma VM and Syrjanen K (1994) Apoptosis in breast cancer as related to histopathological characteristics and prognosis. *Eur J Cancer* **30A**: 2068–2073

Mainwaring PN, Ellis PA, Detre S, Smith IE and Dowsett M (1998) Comparison of in situ methods to assess DNA cleavage in apoptotic cells in patients with breast cancer. *J Clin Pathol* **51**: 34–37

Matter A (1979) Microcinematographic and electron microscopic analysis of target cell lysis induced by cytotoxic T lymphocytes. *Immunology* **36**: 179–190

Potten CS (1996) What is an apoptotic index measuring? A commentary. *Br J Cancer* **74**: 1743–1748

Rich T, Allen RL and Wyllie AH (2000) Defying death after DNA damage. *Nature* **407**: 777–783

Rochaix P, Krajewski S, Reed JC, Bonnet F, Voigt JJ and Brousset P (1999) In vivo patterns of Bcl-2 family protein expression in breast carcinomas in relation to apoptosis. *J Pathol* **187**: 410–415

Sanderson CJ (1976) The mechanism of T cell mediated cytotoxicity. II. Morphological studies of cell death by time-lapse microcinematography. *Proc R Soc Lond B Biol Sci* **192**: 241–255

Sarasin A and Stary A (1997) Human cancer and DNA repair-deficient diseases. *Cancer Detect Prev* **21**: 406–411

Savill J and Fadok V (2000) Corpse clearance defines the meaning of cell death. *Nature* **407**: 784–788

Streuli CH and Gilmore AP (1999) Adhesion-mediated signaling in the regulation of mammary epithelial cell survival. *Journal of Mammary Gland Biology and Neoplasia* **4**: 183–191

Thompson CB (1995) Apoptosis In the Pathogenesis and Treatment Of Disease. *Science* **267 (**n5203): 1456–1462

Vakkala M, Lahteenmaki K, Raunio H, Paakko P and Soini Y (1999) Apoptosis during breast carcinoma progression. *Clin Cancer Res* **5**: 319–324

Vaux DL and Korsmeyer SJ (1999) Cell death in development. *Cell* **96**: 245–254

Wyllie AH, Kerr JF and Currie AR (1980) Cell death: the significance of apoptosis. *Int Rev Cytol* **68**: 251–306

Zhang GJ, Kimijima I, Abe R, Watanabe T, Kanno M, Hara K and Tsuchiya A (1998) Apoptotic index correlates to bcl-2 and p53 protein expression, histological grade and prognosis in invasive breast cancers. *Anticancer Res* **18**: 1989–98

Zheng WQ and Zhan RZ (1998) Quantitative comparison of apoptosis to cell proliferation and p53 protein in breast carcinomas. *Anal Quant Cytol Histol* **20**: 1–6

© 2001 Cancer Research Campaign
